# Singular Case of Ischuria

**Published:** 1808-08

**Authors:** William Parker

**Affiliations:** Charlston, South-Carolina


### *Singular Case of* ISCHURIA; *by Dr.* WILLIAM PARKER, *of Charlston, South-Carolina.*

ON the 4th of November, 1801, Mr. Abraham Markley, a young gentleman of this city, aged eighteen years, usually in high health, and of a habit promising to be muscular, was affected with suppression of urine, accompanied with pain in the lower part of the abdomen, and this particularly violent on any effort to pass urine. For three weeks preceding, he thought he had voided it with some uneasiness, and in a smaller stream than usual. He could assign no cause for these symptoms, though it was ascertained, in the subsequent course of his disease, that he had shortly previous to any affection, exerted himself in climbing up a rope, by the action of his hands and legs, the rope passing in a line down his body, and between his thighs; and further, that he had exercised himself in running and jumping over a fence. His pulse and tongue were in a natural state, the belly slow, appetite not yet impaired, and the urine natural in colour and transparency; nor were there any affections in the neighbourhood of the kidneys.

He took, during the first day, some aperient and diuretic medicines, as also some moderate anodyne doses, and went repeatedly into the warm bath. In the evening, the first of these had operated slightly, but without relief to any of the symptoms. The injections which were given to ensure their passage through the bowels were generally received but partially, and with pain, and for the most part instantly and forcibly rejected. He had voided no urine during the day. The pain in the abdomen was increased. Pressure about the region of the pubes more and more intolerable. Much tension observable along the course of the linea alba, and a slight tumefaction below the umbilicus, inclining to the left; pulse full but soft.

He was now blooded, and the catheter introduced. No resistance in the course of the urethra, and perhaps less than natural at the neck of the bladder. Not more than one ounce of urine, of an ordinary appearance, came off; not, however, without sensible relief. He was directed to continue his medicines, and the bath or fomentations as before, unless ease and consequent sleep should render them inexpedient through the night.

5th. The pain recurred soon after leaving him last evening. No discharge of urine during the night. No rest.

Eight ounces of urine drawn off by the catheter in the morning, and the same quantity at noon. Not more than two ounces came off in the evening. His medicines and other applications through this day the same as yesterday.

*Evening*. Every symptom aggravated; venesection proposed, but not submitted to.

6th. About day-light, after a distressing night, he suffered twenty ounces of blood to be taken away. About eight ounces of urine came off spontaneously, which, in this solitary instance, rendered unnecessary the use of the catheter. At the joint request of Dr. Noble and myself, Dr. Baron was called to visit him at nine, A. M.

Some relaxation of the tension down the linea alba having taken place, a circumscribed tumour could now be clearly felt, reaching above the umbilicus, and extending considerably on each side, but most perceptible on the left. A large blister was applied between the umbilicus and the pubes, and the saline medicines continued through the day.

7th. Has passed some urine in the night, but is now unable to retain it. No other evident relief. Belly bound. Much distressed with pain in the body of the tumour. Took cathartic medicines, which procured some evacuations, and at night the same combined with anodynes.

8th. Costive. Urine evacuated occasionally, though somewhat sparingly. Great distress in the abdomen generally, apparently caused by accumulation of fæces. Injections came away as injected, and were thought to induce a distressing tenesmus from continued repetition. The tumour on the increase.

A cathartic mixture was given at intervals through the forenoon, to no purpose, and in the afternoon strong doses of pills of jalap and calomel. Evacuations followed sufficient to ward off present danger, and to justify the exhibition of moderate anodyne doses, so urgently called for by frequent fits of pain, and inability to sleep. The tumour undiminished, though somewhat less painful on pressure.

9th. The bowels, though not obstructed through this day, yet slow. The purgative medicines continued, and at night again indulged with a moderate anodyne.

10th. A bad night. Fœcal evacuations still continue, but have become remarkably fetid and bilious, accompanied with other appearances leading to apprehend typhous debility. — Urine passed naturally, though rather scantily. The

The tumour rather increased. Took this day his purgative medicines in a light decoction of bark.

11th. Costive. Injections distress him more than ever. Tumour enlarged. Took, through the day, six doses of strong cathartic pills, and sixteen ounces of a cathartic mixture.

12th. No evacuations. Stomach retains nothing; fæces appear diffused with what was ejected. Tobacco smoke, by injection, was now tried without obvious or immediate benefit. Enormous fœcal vomitings during the forenoon exhibited this formidable case in its most shocking form. The warm bath was ordered.

At this crisis, when the attending physicians were about retiring for further consultation, the patient requested that Dr. L. S. a French gentleman in much estimation, might be called to his assistance. The subsequent management of the case devolved principally upon him. On this account, and as the disease soon after became a chronic affection, daily notices of it were omitted.

The case being now considered as *hernia ventralis*, a correspondent treatment was adopted and carried to the utmost possible extent. He continued in the warm bath several hours at a time. Evacuations were solicited by injections, with the cassia fistularis dissolved in them, and by purgatives least apt to irritate. They came on, bringing relief from the vomitings as well as from the general pain in the abdomen. This plan was continued for two or three days, not without sanguine hopes of success; yet it was discouraging to perceive the tumour still on the increase. A tympanitic affection, which was before observable, in a slight degree, soon became the most urgent symptom, and diffused itself over the whole abdomen, presenting an uniform enormous swelling, tense, elastic, and sounding. The warm bath was then desisted from, and tonic, evacuant and carminative medicines again had recourse to, with the effect of removing, in a great measure, the tympanites. Thus the fine constitution of our patient unexpectedly withstood this second shock; though it was only to show us what further variety of suffering an originally good frame, unbroken by intemperance, might endure before it finally yields. On the subsiding of the tympanitic swelling the original tumour came again into view, still larger than before. Several attempts were made to effect a radical cure, according with various views which were at different periods taken of the disease. Among these, after a consultation with Dr. Polony, who first discovered evident fluctuation in the tumour, (by that time grown so large as to come for a considerable extent in contact with the parietes abdominis) tapping was put in practice. On this occasion about half a gallon of *urine* flowed off freely, when, on a sudden, the discharge stopped, and an inexpressible anxiety and pain were diffused over the whole internal abdomen. These, however, gradually lessened during the two or three succeeding days; but at the same time the tumour was reappearing, and on its having gained its first size, his sensations were in their former state. An elegant preparation of the digitalis, prescribed by Dr. Polony, was tried to no purpose; nature began to flag in her resistance to six or eight weeks of unremitted pain, and the want of due action in an important organ; debility and emaciation increased daily: and the treatment was latterly only with a view to palliation.

He was now attacked with a distinct series of inflammatory symptoms, acute pain in the right lumbar region, inability to lie on that side, feverishness, &c. which continued, without being materially relieved, through the remainder of his illness. While these were yet progressing, he perceived, as he thought, something to burst within him, on the left of the tumour, giving him sensible relief; after which a circumscribed part near the spot was very discernible to be much softer than elsewhere. The last affection, in this case, was an inability to move the limbs of the right side, which had continued two days, when death put a period to his miseries.

DISSECTION.

On laying open the abdomen, the bladder presented itself greatly distended, and reaching half way up between the umbilicus and the scrobiculus cordis. Below the umbilicus a strong adhesion existed between it and the peritonæum, by a circular space of about three inches in diameter. In this, however, no inflammatory discolouration remained. Through this adhesion the trocar had passed; of course he was not disturbed by urine in the abdomen. On the left of the bladder, immediately corresponding with the soft spot discovered on the abdomen before death, a protuberance appeared, which being opened, was found to contain about one ounce of urine, in a chamber, formed between the outer and middle coats of the viscus, and communicating with the main cavity by an orifice capable of admitting a small quill. In the latter were found upwards of three quarts of urine, of a common appearance. After it was completely emptied, and wiped with the sponge, it was was soon found to be filling afresh, and this repeatedly; the supply coming from the orifice of the right ureter. While dissecting, with a view to the examination of the kidney, the knife passed suddenly into a large sac, lying adjacent to that viscus, on the right side, containing upwards of a quart of urine. Considerable marks of inflammation appeared in the neighbouring parts, and some pus was lodged at the bottom of the cavity. The kidney itself was paler than usual, and more flaccid; its pelvis (then collapsed) not evidently enlarged, but having a communication between its cavity and that of the sac. The bladder was thickened, and of harder texture than common, though it could not be decidedly pronounced to be in a schirrous state; if by this term, so often applied to diseased bladders be meant an affection of the same nature with schirrous glands.

From these appearances after death, and from the order of the symptoms previous to it, it cannot be doubted that the cyst near the kidney must have been formed by a rupture of the inner coats of its pelvis, and consequent distention of its outer coat: This was a necessary consequence of the bladder's having reached its greatest possible distention, and so resisting the further influx of urine secreted in the kidney. We need only suppose the coats of the bladder here, whether from disease or naturally, stronger than those of the pelvis of the kidney, to account for the rupture not first taking place in the former, as most commonly happens.

This cyst, however, being limited in its distension by the neighbouring parts, while yet the secretion of urine was still going on, it is evident that fresh outlets must have been forced by mere mechanical causes: and thus we find another of the same kind formed upon the bladder itself, and which was on the increase when death occurred.

Divested of these adventitious circumstances, the case resolves itself into some original affection of the bladder. But what this may have been, after all the display of dissection, is perhaps yet matter of mere conjecture.

1. Was it originally a paralysis of the muscular fibres of the bladder, and this accompanied with inflammation of the external coat,* causing the adhesion: and again this

I 3 last

* Inflammation of the one coat, and palsy of the other, are not incompatible; for the same strain or overstretching which would injure the tone of a muscular fibre, applied to an adjoining membrane, must lay the foundation of inflammation in it.

last proving an insurmountable obstacle to any recovery from the paralysis, which the youth of the patient would have entitled him to expect?† It is true, however, that adhesion is not always necessary to such an incurable state of the bladder; as from dissection,‡ similar fatal cases have occurred without adhesion; yet they were in aged subjects, among whom such affections may be expected from the beginning to proceed from bad to worse. If I might hazard an opinion, it would be, that but for the adhesion, a recovery would, in this instance, have taken place. Does Surgery or Physic furnish us with any means of destroying these?

2. In the late stages of these cases, when the patients passed an ordinary quantity of urine, why did not this still continue flowing, seeing the will of the patient coincided? No obstruction existed about the neck of the bladder or in the urethra, and the pressure of the neighbouring viscera, as well as that from much condensed air, must have been considerable.

3. Would the continued use of the catheter be of any avail in such cases? It is regretted that our patient would not submit to the trial of it, which was actually proposed after the perusal of the cases quoted above. Yet it could scarcely have failed to excite fresh distress at the place of adhesion: nor does former observation favour its use but as a palliative. A man in the Pennsylvania Hospital, affected with inability to pass urine from a fall, had it drawn off daily for three months, at the expiration of which time he died. The bladder was found hard, contracted, and thickened.

---

† It is certain that the adhesion would prevent the natural and full contraction of the muscular fibres, which, thus kept for a long time over-distended, might lose altogether their disposition to it. Early in the disease, the patient said, that, on attempting to pass urine, he felt as if a wall was placed against his bladder, evidently a sensation arising from the adhesion resisting contraction.

‡ Vide Encyclopædia, Art. *Ischuria vesicalis.*

OBSERVATIONS

